# Editorial: Impact of blood flow restriction device features and methodological considerations on acute- and longitudinal responses to blood flow restricted exercise

**DOI:** 10.3389/fspor.2026.1807687

**Published:** 2026-03-06

**Authors:** Nicholas Rolnick, Tim Werner, Matthew Clarkson, Luke Hughes

**Affiliations:** 1 The BFR PROS, New York City, NY, United States; 2 Department of Exercise Science, Salisbury University, Salisbury, MD, United States; 3 Institute for Health and Sport, Victoria University, Melbourne, VIC, Australia; 4 Department of Sport, Exercise and Rehabilitation, Northumbria University, Newcastle upon Tyne, United Kingdom

**Keywords:** autoregulation, interface pressure, Kaatsu, multi-chambered cuffs, bstrong, BFR training

Blood flow restriction (BFR) training has expanded rapidly across rehabilitation, sport performance, and general fitness settings due to the meaningful adaptations it elicits using lower external intensities of exercise, often comparable to high intensity training. As a byproduct of the growth of BFR, various device systems are available to the consumer, many of which may differ in material, bladder design, width, and ability to apply a consistent pressure to the exercising limb. All of these factors have been either theorized or shown to have the capacity to impact the acute BFR training stimulus, and therefore, should not be ignored in the study or prescription of BFR training. This Research Topic aimed to clarify how BFR device design and pressure-prescription methodology shape the BFR stimulus and downstream outcomes in healthy, athletic, and clinical populations. The contributions broadly fit into three themes: 1) device features and pressure personalization as central determinants of the BFR stimulus 2) cardiovascular and autonomic responses, safety and special populations 3) meta-analyses investigating dose-response and efficacy.

As device features continue to evolve for use in BFR research and practice, it is critical for researchers and clinicians to understand how the existing literature supports – or fails to support – device-specific responses to BFR training. Rolnick reported that multi-chambered bladder cuff designs largely prevent meaningful arterial occlusion and therefore cannot reliably individualize applied pressure relative to an individual's arterial occlusion pressure (AOP). Consequently, these cuffs may lead to systematic overestimation of the effective pressure experienced by participants when implemented in research settings or in those attempting to determine a pressure-dependent relationship on physiological outcomes such as cortical activation and cerebral oxygenation (Rolnick et al.). This limitation becomes particularly problematic when studies attempt to relativize pressure using limb-circumference-based algorithms developed for single-chambered cuffs, potentially producing a BFR stimulus that is substantially lower than intended.

To illustrate the potential differences in acute physiologic stress associated with multi-chambered cuffs, Rolnick et al. compared fatiguability, central stiffness responses, and perceptual demands during lower-body BFR exercise with a single-chambered BFR cuff inflated to 60% supine AOP vs. a multi-chambered bladder inflated to 300 mmHg. The findings demonstrated that bladder design materially alters performance, perceptual, and vascular responses to BFR exercise and should be considered in BFR methodologies. Stray-Gundersen and Stampley further discuss nuances associated with this multi-chambered cuff design in a narrative review and provide practical application guidelines informed by the existing body of research utilizing these systems.

Rolnick additionally describes how autoregulation of applied BFR pressures by the device system itself may alter the acute BFR stimulus, drawing on divergent exercise performance outcomes observed in cohorts training with two different autoregulating BFR cuffs. Importantly, the accompanying editorial cautions against making blanket statements regarding autoregulation, as the limited available evidence suggests that responsiveness may vary, thereby influencing acute physiological and performance responses to BFR exercise. Ogrezeanu et al. added clinical context by comparing autoregulated and non-autoregulated BFR at 50% AOP in patients with hemophilia. Their findings showed trends towards more favorable acute cardiovascular and neuromuscular responses, including hypotensive and hypoalgesic effects with autoregulated BFR pressures, indicating that there may be benefit to implementing autoregulation when applying BFR within this cohort of patients.

Finally, Rolnick et al. describe how the pressure set for BFR exercise does not always equal the pressure applied at the limb-cuff interface and outline the potential ramifications of this discrepancy for interpreting and implementing BFR training. Swain et al. provided empirical data on four commercially available BFR cuffs and reported that most were unable to consistently maintain target pressure during exercise, with only the Delfi Personalized Tourniquet System maintaining set pressure within ±10% for most of the exercise bout. Further, despite targeting 80% AOP, some cuffs transiently applied pressures exceeding 100% AOP for up to 55% of the set, highlighting device-specific safety and dosage concerns.

Bommasamudram et al. compared agreement between five different BFR devices for AOP values in supine and standing positions and found that no device demonstrated consistent agreement with the Zimmer surgical-grade cuff. However, posture-dependent changes in AOP underscore the need to consider body position when assessing and prescribing AOP. Lastly, Zhu et al. investigated the impact of varying percentages of AOP on muscle activation, post-exercise blood lactate, and perceptual demands. Their findings suggest pressure-dependent changes with increasing applied pressures and indicate that moderate pressures (∼70% AOP) may elicit comparable physiological responses to higher pressures while imposing lower perceived exertion.

Rolnick et al. provided critical commentary on a study with insufficient reporting of BFR methodology, emphasizing that when investigating the effects of pressure on physiological responses, comprehensive device reporting and appropriate study design are essential for valid interpretation.

Other studies within this Research Topic examined safety screening and pressure application, autonomic responses to varying pressures, cardiovascular responses in animal models, the integration of BFR with variable resistance training, and multiple systematic reviews and meta-analyses. Collectively, these works help contextualize how BFR and BFR-related methodological decisions influence both acute physiological responses and chronic training adaptations.

Wedig et al. reported on a web-based BFR implementation tool designed to assist practitioners with safety screening and reduce barriers to clinical and applied use. The authors observed high usability with minimal errors and reported that users demonstrated greater confidence in BFR and an increased likelihood of implementation when using the tool, largely because it addressed previously identified barriers related to screening, equipment selection, and pressure determination (1). Additionally, Wedig et al. investigated predictors of lower-limb AOP across cuffs of different widths to develop cuff-specific prediction equations for practitioners who lack access to Doppler ultrasound or other instrumentation capable of directly determining AOP.

Garner et al. implemented intermittent BFR pressure application at 20% 1RM during leg extension exercise and observed reduced vastus lateralis muscle oxygenation and increased ratings of perceived exertion compared with control exercise, indicating greater local metabolic stress. However, post-exercise autonomic modulation did not differ indicating that intermittent BFR application did not induce additional autonomic disturbances and may be a suitable approach to strengthening in those with clinical considerations that cannot perform continuous based BFR pressure application. Peng et al. showed that an 8-week intervention where BFR was combined with chain-based variable resistance significantly improved lower-limb strength, jump performance, sprint speed, and thigh circumference in collegiate basketball players comparable to high-load chain-based variable resistance. Their findings provide continual expansion of the contexts in which low load BFR can be an appropriate substitute for higher load resistance exercise. In an animal model, ShangGuan et al. investigated the impact of low-intensity BFR training on cardioprotective molecular profiles and found that low intensity BFR may have some protective effect on the myocardium.

Last, four systematic reviews and meta-analyses provide additional context demonstrating that the physiological and performance effects of BFR are highly contingent on pressure, training load, frequency, and population. Wang et al. demonstrated that upper-limb BFR acutely enhances strength while substantially increasing fatigue, with the largest immediate effects occurring when moderate-to-high loads (40%–70% 1RM) are paired with higher relative pressures (≥60% AOP), underscoring a clear pressure-load interaction for acute responses. Su et al. extended these findings to athletic training, showing that combining BFR with high-load resistance training produces superior improvements in strength, power, speed, and endurance compared with high-load training alone, particularly during higher-frequency applications (≥3 sessions/week). They also reported that absolute and individualized pressure strategies yield broadly similar strength outcomes but may produce divergent performance effects, emphasizing the potential importance of pressure personalization for balancing safety and effectiveness in applied and clinical settings.

In older adults, Ren et al. further refined this dose-response framework by concluding that high-frequency, high-pressure, low-intensity BFR is most favorable for isometric strength and hypertrophy, whereas lower-frequency, lower-pressure approaches preferentially improve 1RM and may influence blood pressure, highlighting that protocol selection carries distinct neuromuscular and cardiovascular implications. Finally, Kong et al. situated BFR within a broader health context, reporting modest reductions in systolic blood pressure and body fat percentage without meaningful changes in aerobic capacity or body mass, suggesting that BFR may be best positioned as a complementary adjunct to training rather than a primary conditioning stimulus.

Collectively, this special topic collection highlights the myriad ways in which the BFR stimulus can be applied and how these choices influence both acute and chronic training responses. As BFR continues to grow, researchers and practitioners should be critical of cuff-specific features, and emphasize methodological rigor and comprehensive reporting to enhance generalizability and improve the overall quality of the expanding BFR evidence base.
